# Inflammatory markers from routine blood tests predict survival in multiple myeloma: a Systematic Review and meta-analysis

**DOI:** 10.3389/fimmu.2025.1669878

**Published:** 2025-10-30

**Authors:** Mengjiao Luo, Ling Qin, Yujie Li, Qianru Mei, Qiaoping Wu, Xudong Feng

**Affiliations:** Department of Clinical Laboratory, Ningbo Medical Center Lihuili Hospital, The Affiliated Lihuili Hospital of Ningbo University, Ningbo, China

**Keywords:** multiple myeloma, NLR, LMR, RDW, prognosis

## Abstract

**Background:**

Multiple myeloma (MM) is an incurable hematologic malignancy marked by abnormal plasma cell proliferation. Inflammatory indices derived from routine blood tests—such as neutrophil-to-lymphocyte ratio (NLR), lymphocyte-to-monocyte ratio (LMR), platelet-to-lymphocyte ratio (PLR), red cell distribution width (RDW), RDW-to-platelet ratio (RPR), and hemoglobin-to-RDW ratio (HRR)—have shown prognostic value across cancers. This meta-analysis aimed to evaluate their prognostic significance in MM.

**Methods:**

Following PRISMA guidelines, a systematic search of PubMed, Embase, and Web of Science identified eligible studies through January 17, 2025. Pooled hazard ratios (HRs) and odds ratios (ORs) with 95% confidence intervals (CIs) were calculated. Sensitivity and subgroup analyses were conducted to assess heterogeneity, and publication bias was evaluated using Egger’s and Begg’s tests.

**Results:**

Twenty-seven studies including 5,009 MM patients were analyzed. Elevated NLR was significantly associated with poor overall survival (OS: HR = 2.06, 95% CI: 1.72–2.47) and progression-free survival (PFS: HR = 1.70, 95% CI: 1.32–2.19), as well as advanced disease stage (OR = 2.85, 95% CI: 1.40–5.80). High RDW and low LMR were similarly linked to worse outcomes (RDW–OS: HR = 1.68; LMR–OS: HR = 0.58). PLR showed no significant association with prognosis. RPR and HRR results were inconsistent due to limited data.

**Conclusion:**

NLR, LMR, and RDW are promising prognostic biomarkers in MM, with elevated NLR and RDW and decreased LMR indicating poorer outcomes. PLR, RPR, and HRR require further investigation. These routinely accessible indices may aid in clinical risk stratification and therapeutic decision-making.

**Systematic review registration:**

https://www.crd.york.ac.uk/PROSPERO/view/CRD420251105106, identifier CRD420251105106.

## 1 Introduction

Multiple myeloma (MM) is an incurable malignant tumor caused by abnormal proliferation of B cells, primarily affecting the bone marrow. It presents with complex clinical manifestations, including bone destruction, suppression of marrow function, and renal failure (1). As the second most common hematologic malignancy in many countries (2), MM requires timely intervention to improve patient survival and quality of life. Therefore, the identification of useful, easily accessible, and cost-effective biomarkers is of great importance.

Inflammatory biomarkers serve as a critical bridge linking systemic immune status with tumor prognosis, holding significant potential for clinical translation. A growing body of evidence suggests that systemic inflammation plays an important role in tumor development (3). Elevated neutrophil-to-lymphocyte ratio (NLR) has been consistently reported to be associated with poor prognosis in a variety of solid tumors—such as lung (4, 5), gastric (6, 7), colorectal (8, 9), and liver cancers (10, 11)—as well as in hematologic malignancies including lymphoma (12, 13). Similarly, the lymphocyte-to-monocyte ratio (LMR) has been identified as an independent prognostic factor in cancers such as prostate (14), gastric (15), and lung (16). The prognostic value of the platelet-to-lymphocyte ratio (PLR) has also been validated in gastric cancer (17, 18), glioblastoma (19), and hepatobiliary carcinoma (20), among others. Moreover, red cell distribution width (RDW) is increasingly recognized as an independent prognostic marker in multiple cancer types, including lung (21, 22), breast (23, 24), and colorectal cancers (25, 26). Additionally, both the RDW-to-platelet ratio (RPR) and the hemoglobin-to-RDW ratio (HRR) have been reported to provide valuable prognostic information in cancer patients (27–29).

Although several studies have investigated blood-derived inflammatory indicators in MM, their prognostic value remains inconclusive due to inconsistent results across studies. Meta-analysis, by integrating data from different cohorts and minimizing the limitations of individual studies, provides more reliable and precise effect estimates. Therefore, this study aimed to systematically evaluate the prognostic significance of NLR, LMR/MLR, PLR, RDW, RPR, and HRR in MM patients.

## 2 Methods

### 2.1 Search strategy

This meta-analysis and systematic review followed the Preferred Reporting Items for Systematic Reviews and Meta-Analyses (PRISMA) guidelines (30), and the analysis protocol was prospectively registered in PROSPERO (CRD420251105106). We conducted a comprehensive literature search of PubMed, Embase, and Web of Science databases, with the last search performed on January 17, 2025. The following search terms and their combinations were used: (NLR OR neutrophil-to-lymphocyte ratio), (LMR OR lymphocyte-to-monocyte ratio OR MLR OR monocyte-to-lymphocyte ratio), (PLR OR platelet-to-lymphocyte ratio), (RDW OR red blood cell distribution width OR red cell distribution width), (RPR OR RDW-to-platelet ratio OR red cell distribution width-to-platelet ratio), (HRR OR hemoglobin-to-red blood cell distribution width ratio OR hemoglobin-to-RDW ratio), and (MM or Multiple Myeloma or Kahler disease or Myelomatosis or plasma cell myeloma). In addition, the reference lists of all relevant publications were manually screened to identify any additional eligible studies.

### 2.2 Study selection

Two investigators (Mengjiao Luo and Ling Qin) independently screened all identified articles for eligibility, with discrepancies resolved through discussion or adjudication by a senior researcher. The selection process adhered to the following predetermined criteria (1): patients were diagnosed with MM by the most recent diagnostic criteria (2), reported the relationship between pre-treatment indicators and MM prognosis (3), reported survival outcomes with either, Directly reported hazard ratios (HRs) and corresponding 95% confidence intervals (CIs), Sufficient data to calculate HRs and 95% CIs or Kaplan-Meier curves amenable to digital extraction and reconstruction (4), full-text articles published in English.

### 2.3 Data extraction and quality assessment

The data obtained from the studies included in this analysis encompassed a range of variables, such as (1): Study characteristics: First author, publication year, country of origin, (2) participant demographics: Sample size, sex distribution, age range, follow-up duration, (3) prognostic indicators: Cutoff values, International Staging System (ISS) classification, HRs with 95% confidence intervals, Newcastle-Ottawa Scale (NOS) scores.

Study quality was rigorously assessed using the validated NOS for cohort studies (31). The NOS score consists of three components: subject selection (0–4 points), between-group comparability (0–2 points), and outcome assessment (0–3 points). Studies achieving total NOS scores ≥ 7 were classified as high-quality, indicating robust methodology with minimal risk of bias.

### 2.4 Data synthesis and analysis

The prognostic data extraction and analytical methodology were systematically implemented as follows: HRs with corresponding 95% CIs were extracted directly from study reports when available, while for studies providing only survival curves, we utilized Engage Digitizer to digitally reconstruct time-to-event data and derive HR estimates. All statistical analyses were conducted using Review Manager 5.4 and STATA 14.0, with both packages employed for data visualization. Between-study heterogeneity was quantitatively assessed using the I² statistic, with values exceeding 50% considered indicative of substantial heterogeneity, thereby determining model selection – employing the random-effects model for I² > 50% or the fixed-effects model for I² ≤ 50%. Sensitivity analyses incorporated a leave-one-out approach to evaluate the robustness of pooled estimates and identify potential outlier studies, while prespecified subgroup analyses examined potential heterogeneity sources across study characteristics (design, sample size), patient demographics (age distribution, disease stage), and methodological factors (assay methods, cutoff values). Publication bias was comprehensively evaluated through both visual inspection of funnel plot asymmetry and formal statistical testing using Egger’s linear regression and Begg’s rank correlation tests, with p-values < 0.10 considered suggestive of potential bias.

## 3 Results

### 3.1 Literature search and study characteristic

**Figure 1:**
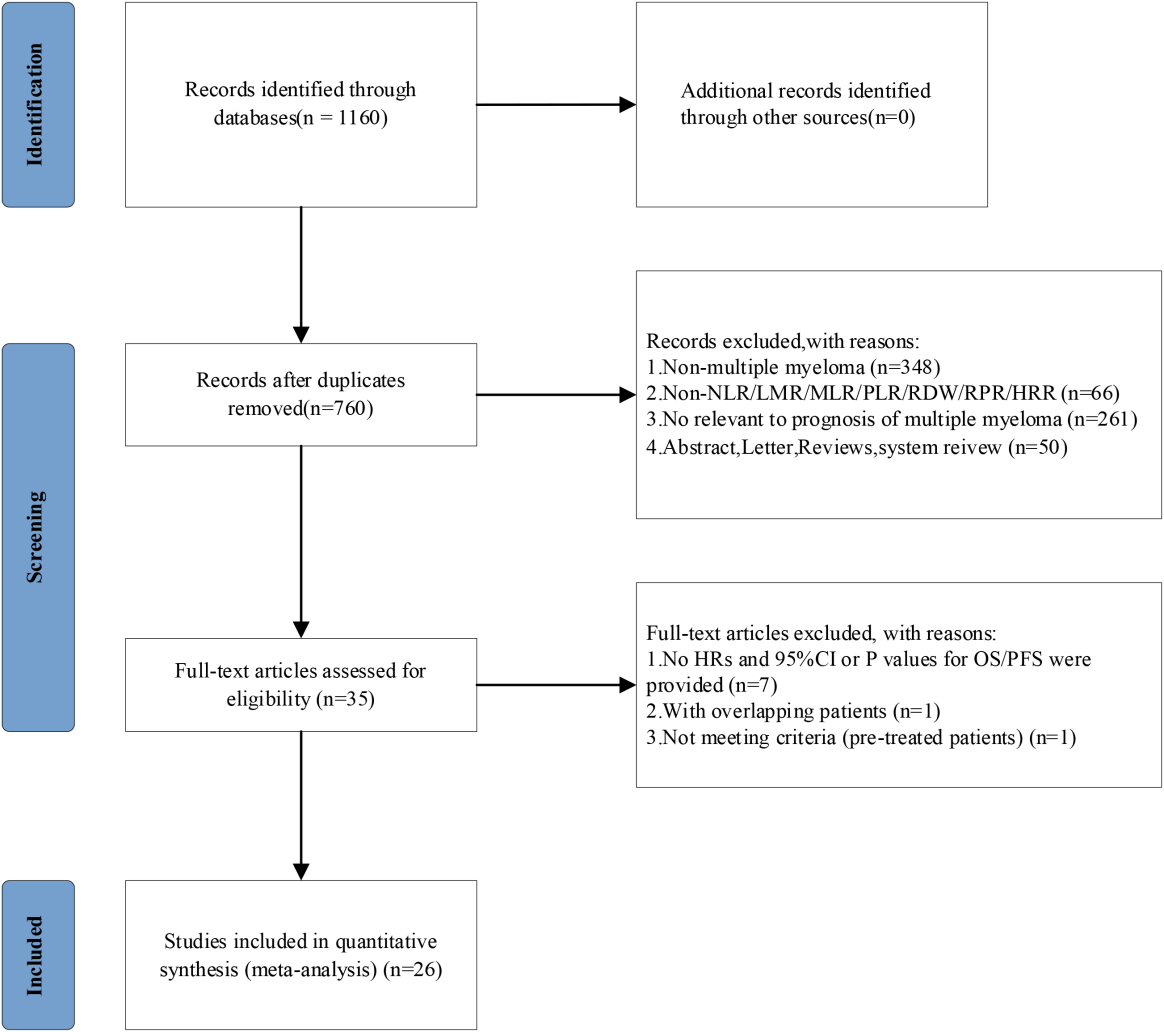
PRISMA flow chart of the study selection procedure.

The study selection process (**Figure 1**) employed a rigorous multi-stage screening methodology in strict accordance with PRISMA guidelines. Our systematic search of PubMed, Embase, and Web of Science initially identified 1,160 potentially relevant publications, from which 400 duplicate records were removed through combined automated and manual processes. Subsequent dual-independent title/abstract screening excluded 725 publications, including 348 non-MM studies, 66 articles lacking pertinent hematological indices, 50 ineligible publication types (animal studies, conference abstracts, systematic reviews), and 261 studies not addressing MM prognostic outcomes. The remaining 35 articles underwent comprehensive full-text evaluation, resulting in the final inclusion of 26 studies that met all predefined eligibility criteria, encompassing publications from 2013 to 2024 with explicit reporting of prognostic hematological indices in MM. Throughout this process, all exclusion decisions were meticulously documented and cross-verified by two independent reviewers, with any discrepancies resolved through consensus discussion involving a third investigator.

### 3.2 Study characterization and quality assessment

The key characteristics of the 26 studies analyzed in this paper (32–57) are shown in **Table 1**. The included studies were retrospective cohort studies except for one study (40), which was prospective and included a total of 4909 patients. The mean age of the study population ranged from 60 to 67 years, with males accounting for 53% to 59% of the patients in the survey dataset. Twelve of these studies (33, 35–41, 44, 46, 47, 52) (2459 patients) reported the prognostic correlation between NLR and MM patients, eight studies (34, 39, 40, 42, 45, 48, 49, 55) (2041 patients) on the prognostic relationship between LMR/MLR and MM patients, six studies (33, 36, 40, 41, 44, 56) (1510 patients) on the prognostic relationship between PLR and MM patients, seven studies (33, 37, 43, 50, 51, 53, 54) (1141 patients) on the prognostic relationship between RDW and MM patients, two studies (32, 57) (255 patients) on the prognostic relationship between RPR and MM patients, and one study (55) (180 patients) on the prognostic relationship between HRR and MM patients. Eighteen of the studies (32, 33, 35–37, 40, 42, 45, 46, 48–54, 56, 57) (3381 patients) had Asian participants, 14 reported (32–35, 37, 38, 44–46, 49–51, 56, 57) (2251 patients) HR directly, and 12 (36, 39–43, 47, 48, 52–55) (2658 patients) calculated HR via survival plots. Although the study data were all collected pre-treatment, cutoffs for the relevant metrics (NLR, LMR, PLR, RDW, RPR) varied across studies and were obtained using a variety of methods. Quality studies were assessed on the Newcastle-Ottawa Scale on a scale of 7 to 9, indicating that the survey methodology was generally of good quality with a low risk of bias.

**Table 1:** Baseline characteristics of included studies.

Study	Year	Country	Number (M/F)	Age (months)	Follow-up (months)	ISS stage (n)	Cut-off value						Outcome	HR	NOS score
							NLR	LMR	PLR	RDW	RPR	HRR			
Lu WG (32)	2023	China	110 (58/52)	61 (31-84)	36	I/II/III (21/41/48)					NR		OS	M	7
Meng S (33)	2018	China	166 (88/78)	62 (34-93)	18.48 (0.90-62.83)	NR	1.97		98.45	14%			OS\PFS	M	8
Dosani T (34)	2017	USA	372 (196/176)	67.3 (30-92)	37.5 (1.16-152.9)	I/II/III (97/170/78)		3.6					OS\PFS	M	7
Kim DS (35)	2017	Korea	273 (160/113)	64 (30-83)	NR	I/II/III (56/110/107)	2.25						OS	M	7
Li YJ (36)	2016	China	315 (196/119)	NR	25 (1-64)	I/II/III (43/125/147)	2		119				OS\PFS	U	8
Liu SW (37)	2019	China	175 (95/80)	61	33.63 (2.17-79.33)	I/II/III (23/44/108)	2			14%			OS	M	8
Onec B (38)	2017	Turkey	52 (28/24)	65.5 (34-88)	35.1	I/II/III (7/18/27)	1.72						OS	M	7
Romano A (39)	2017	Italy	208	58 (31-66)	36	I/II/III (54/77/77)	2	3.6					PFS	U	9
Shi LH (40)	2017	China	560 (344/216)	NR	64	I/II/III (100/195/265)	4	MLR-0.3	100				OS\PFS	U	7
Solmaz S (41)	2018	Turkey	186 (104/82)	60 (29–89)	44 (2-146)	I/II/III (45/52/64)	1.9		120				OS\PFS	U	7
Tian Y (42)	2018	China	285 (159/126)	NR	48 (2-84)	NR		4.2					OS\PFS	M	7
Yigit Ayhan E (43)	2024	Turkey	218 (120/98)	61.55	NR	I/II/III (36/70/112)				16.5%			OS\PFS	U	7
Wongrakpanich S (44)	2016	USA	161 (81/80)	69 (41-91)	NR	I/II/III (46/61/34)	2.78		155.58				OS	M	7
Yang Y (45)	2020	China	102 (67/35)	NR	14.23 (0.17-60.4)	I/II/III (5/36/61)		3.7					OS	M	8
Zhou X (46)	2018	China	76 (41-35)	63 (40-79)	34 (1-93)	I/II/III (3/35/38)	2.95						OS	U	8
Kelkitli E (47)	2014	Turkey	151 (83/68)	63 (35-89)	41	I/II/III (23/54/74)	2						OS	U	7
Zhang XY (48)	2016	China	145 (78/67)	NR	27 (2-96)	I-II/III (106/39)		2.9					OS	U	7
Shin SJ (49)	2013	Korea	189 (98/91)	60 (29-84)	31.27 (0.07-167.0)	I/II/III (35/87/61)		2.9					OS	M	7
Zhou D (50)	2018	China	162 (87/75)	61 (40-87)	NR	I/II/III (35/67/60)				14%			OS\PFS	M	7
LEE H (51)	2014	Korea	146 (91/55)	61 (32-83)	47 (3-104)	I/II/III (60/49/35)				14.5%			PFS	M	8
Zuo HQ (52)	2017	China	136 (73/63)	61 (40-80)	27	I/II/III (14/106/16)	2						OS\PFS	M	8
Ma YY (53)	2018	China	78 (47/31)	60.7 (43-81)	42.6 (2-136)	I-II/III (51/27)				15.5%			OS\PFS	M	8
Wang J (54)	2017	China	196 (110/86)	65 (33-82)	33.5 (1-120)	I/II/III (6/69/71)				18.05%			OS	M	8
Baysal M (55)	2020	Poland	180 (87/93)	66.77 (28-93)	NR	I/II/III (68/51/61)		3.28				0.61	OS	M	8
Zhang QE (56)	2025	China	122 (61/61)	63 (30-77)	34 (1–146)	I-II/III (63/59)			76.25				OS\PFS	M	8
Li MY (57)	2023	China	145 (79/66)	59 (38-85)	NR	I-II/III (86/58)					0.12		OS\PFS	M	8

NR, not reported; OS: overall survival; PFS, progression-free survival; HR risk ratio, M, multivariate analysis, U, univariate analysis, NOS, Newcastle-Ottawa Quality Scale.

### 3.3 Prognostic value of neutrophil-to-lymphocyte ratio in multiple myeloma

#### 3.3.1 Prognostic value of NLR in multiple myeloma: preliminary findings

**Figure 2:**
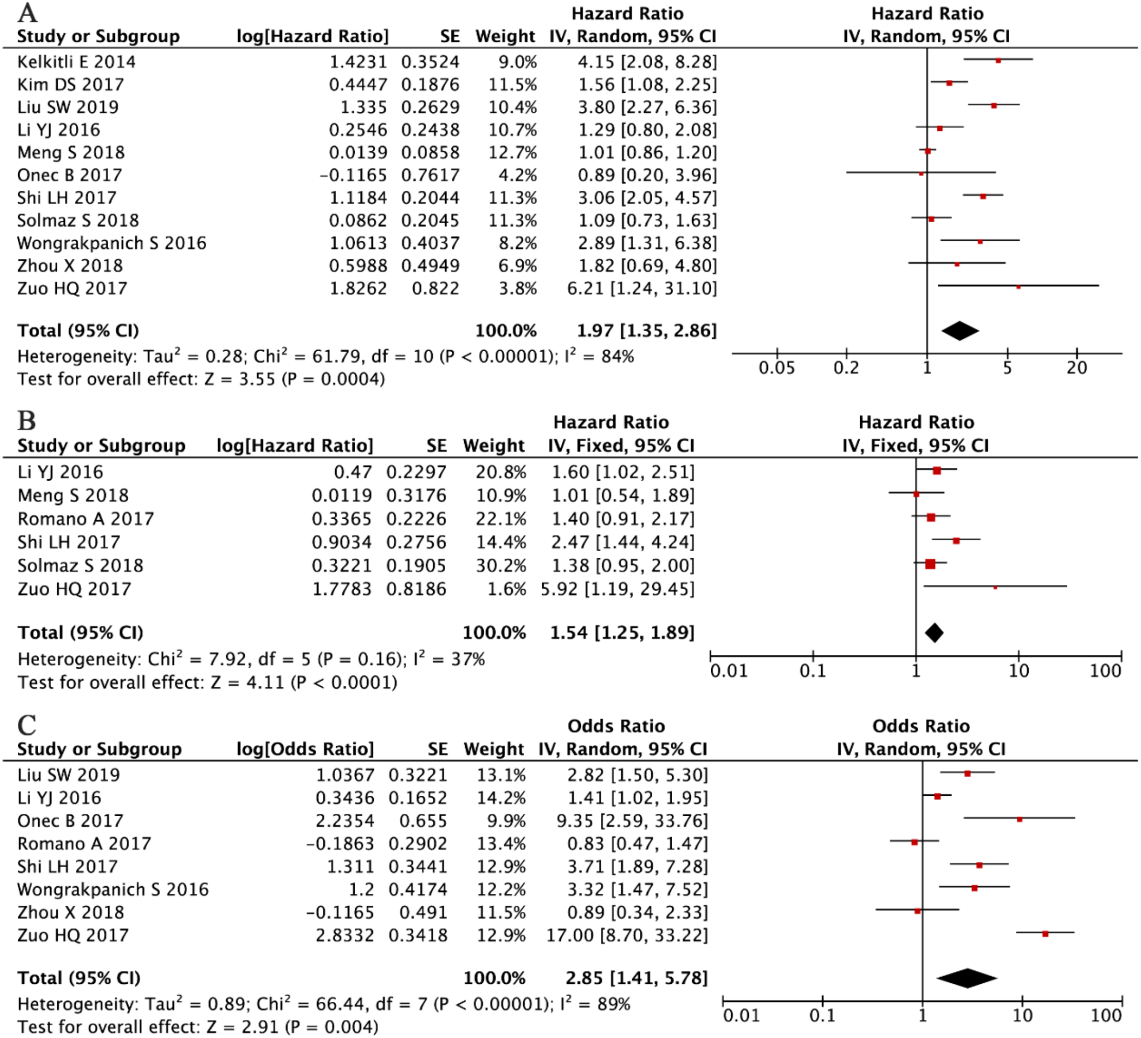
Forest plots illustrating the prognostic significance of NLR in multiple myeloma. **(A)** Overall survival. **(B)** Progression-free survival. **(C)** Association between NLR and disease stage according to the International Staging System.

The prognostic significance of NLR in MM was assessed by pooling data from 11 clinical studies involving 2,351 patients (33, 35–38, 40, 41, 44, 46, 47, 52). A random-effects meta-analysis, warranted by substantial between-study heterogeneity (I² = 84%, P < 0.001), revealed a significant association between elevated NLR levels and poorer OS (HR = 1.97, 95% CI: 1.35-2.86, P < 0.001; **Figure 2A**). Among these studies, eight provided multivariate analyses, while four reported univariate analyses.

Consistent with these findings, the PFS analysis, incorporating data from six studies with a total of 1,571 patients (34, 37, 40–42, 53), demonstrated that elevated NLR was significantly associated with poorer PFS outcomes. Given the moderate and non-significant heterogeneity among studies (I² = 37%, P = 0.16), a fixed-effects model was applied (HR = 1.54, 95% CI: 1.25–1.89, P < 0.001; **Figure 2B**). Furthermore, analysis of clinicopathological associations across eight independent cohorts (n = 1,683) (36–40, 44, 46, 52) demonstrated that higher NLR values were strongly correlated with advanced disease stage (ISS III versus I-II: OR = 2.85, 95% CI: 1.41-5.78, P = 0.004; **Figure 2C**). These consistent findings across different clinical endpoints reinforce the role of NLR as a reliable prognostic indicator in multiple myeloma.

#### 3.3.2 Sensitivity analysis of NLR for overall survival

**Figure 3:**
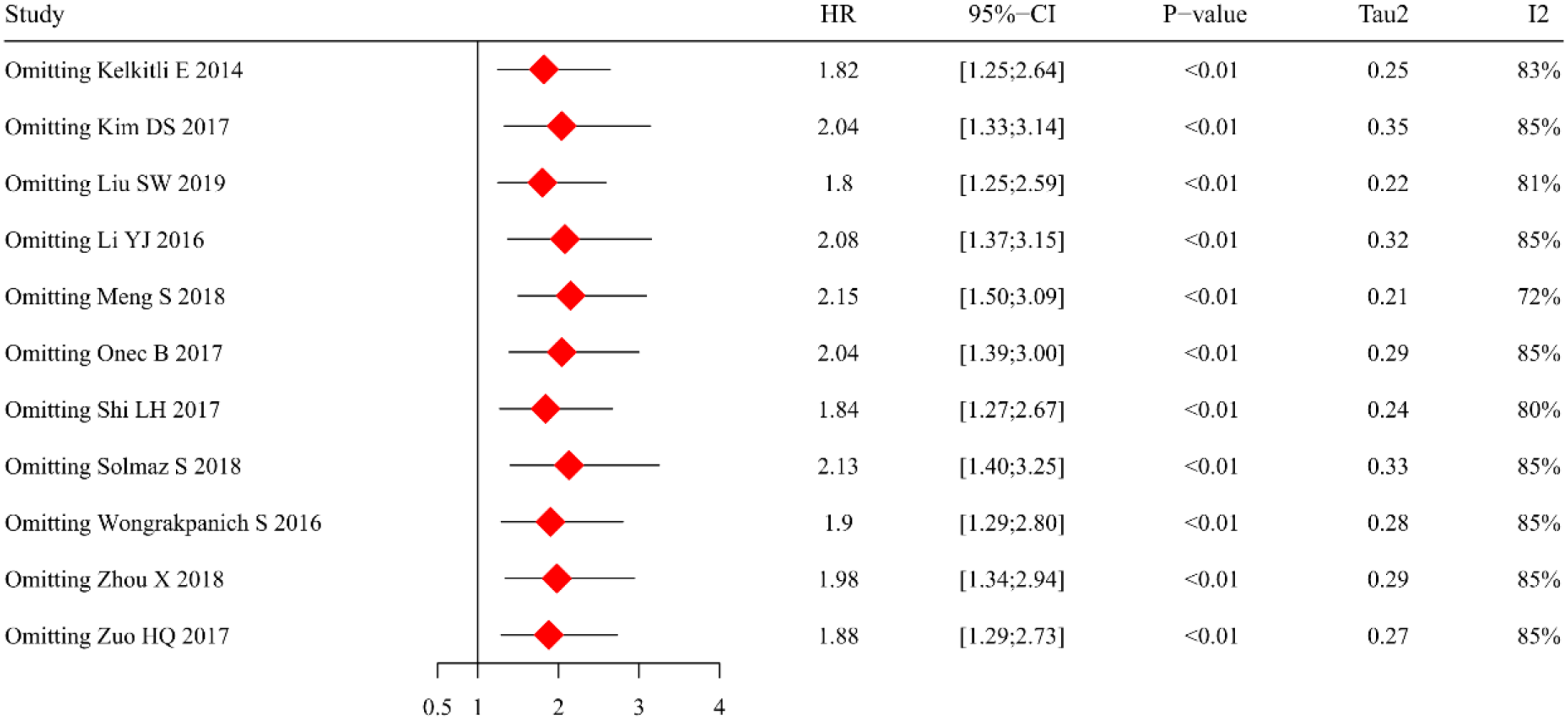
Leave-one-out sensitivity analysis of the effect of NLR on OS.

The sensitivity analysis was conducted to assess the robustness of our findings and to elucidate potential sources of heterogeneity. Using a random-effects model approach, we performed sequential exclusions of individual studies to evaluate their impact on the pooled HR estimates (**Figure 3**). Although the effect size showed some variability across these iterations, the primary observation that elevated NLR predicts inferior OS remained consistently significant. Of particular note, the study by Meng et al. (33) was identified as a substantial source of heterogeneity (I² = 84%). Subsequent exclusion of this study resulted in a marked 72% reduction in heterogeneity, while preserving the statistically significant association between increased NLR levels and poorer OS outcomes.

#### 3.3.3 Re-analyze the prognostic value of NLR by comprehensively synthesizing the included studies

**Figure 4:**
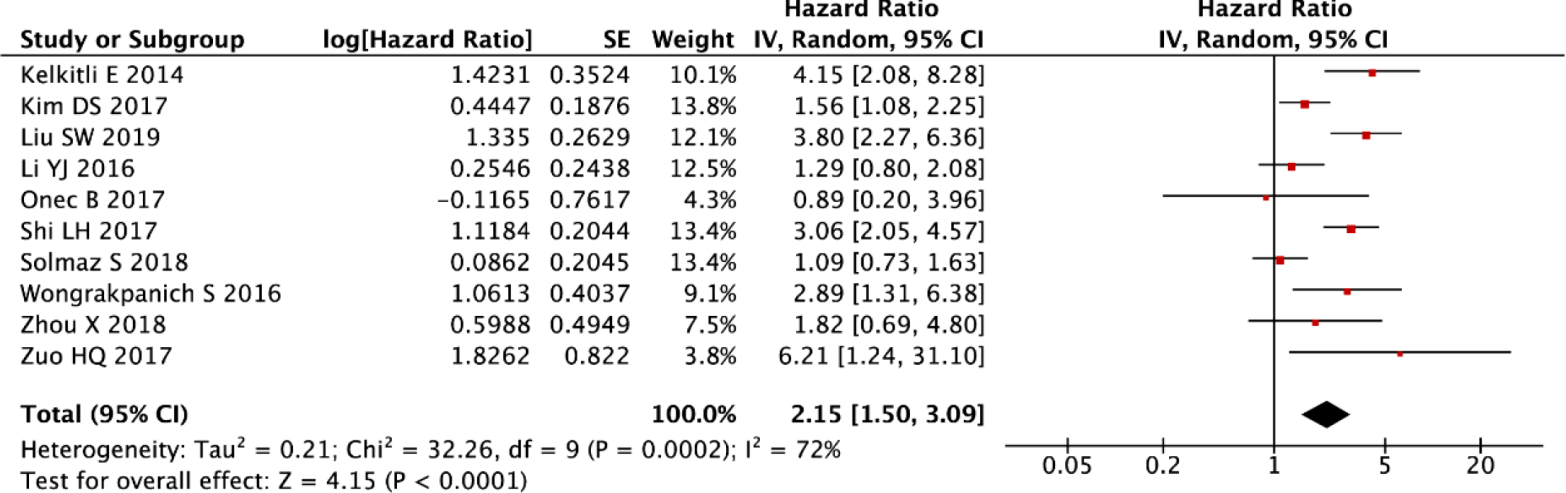
Forest map resynthesized after sensitivity analysis to determine exclusion of a study.

The post-sensitivity evaluation demonstrated robust prognostic validity of NLR in multiple myeloma, with persistently elevated NLR levels predicting significantly worse OS (random-effects model: HR = 2.15, 95% CI: 1.50-3.09, P < 0.001; **Figure 4**) following the exclusion of outlier studies. While this refined analysis substantially reduced initial heterogeneity, moderate residual variability was still observed (I² = 72%, P < 0.001), as shown in **Figure 4**. These findings collectively reinforce NLR as a stable prognostic indicator, with the residual heterogeneity likely reflecting clinically meaningful variations in patient populations or treatment protocols across studies.

**Figure 5:**
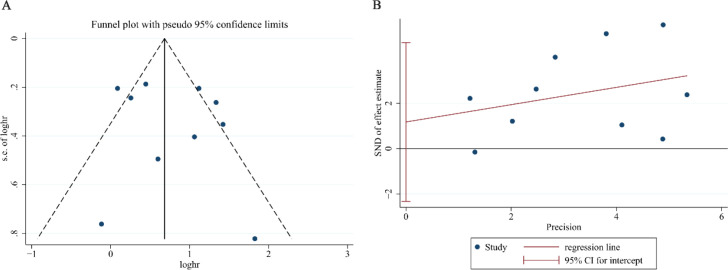
Assessment of publication bias for NLR overall survival after exclusion of the study by Meng S. **(A)** Funnel plot for detecting potential publication bias. **(B)** Egger’s regression test for statistical evaluation of publication bias.

We evaluated publication bias for the re-analyzed OS data through both graphical and statistical methods. Visual inspection of the funnel plot (**Figure 5A**) revealed a symmetrical distribution of study estimates, while formal statistical tests confirmed the absence of significant publication bias (Egger’s test: P = 0.462, **Figure 5B**; Begg’s test: P = 0.858), thereby providing robust evidence that no substantial publication bias was present in our meta-analysis.

**Figure 6:**
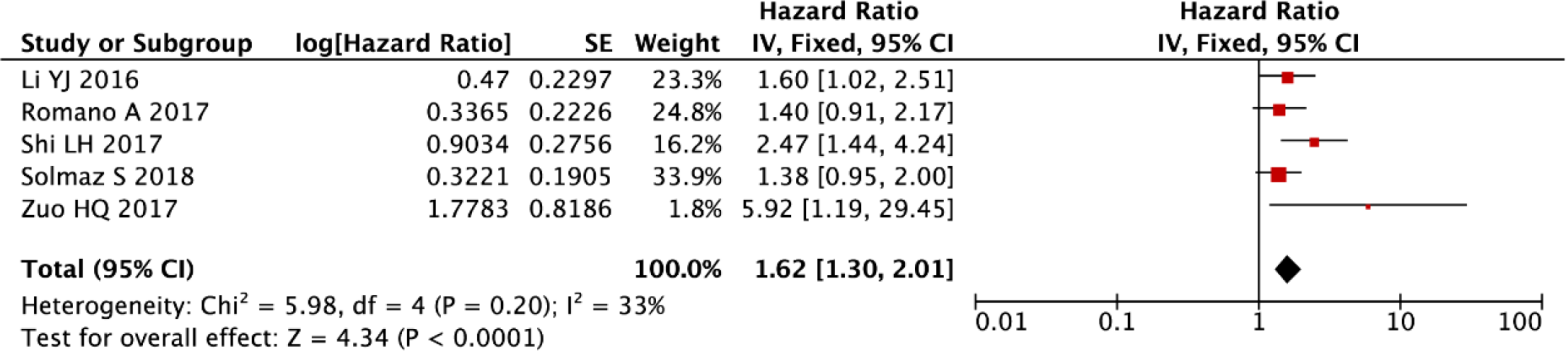
Forest plot of NLR for PFS after exclusion of Meng S.

After rigorous exclusion of influential outliers, our refined meta-analytic approach substantiated that elevated NLR retains significant prognostic value for PFS in multiple myeloma (fixed-effect model: HR = 1.62, 95% CI: 1.30-2.01, P < 0.001), with these robust findings illustrated in **Figure 6**. The consistency of this association across sensitivity analyses underscores NLR’s reliability as a hematologic prognostic marker.

#### 3.3.4 Subgroup analysis of NLR for overall survival

Our meta-analysis identified substantial between-study heterogeneity in the association between NLR and OS (I² = 84%, P < 0.001). To elucidate potential modifiers of this heterogeneity, we performed prespecified subgroup analyses of 10 eligible studies (35–38, 40, 41, 44, 46, 47, 52), systematically evaluating five clinically relevant stratifications: (a) statistical methodology (univariate versus multivariate modeling), (b) geographic distribution (Asian versus non-Asian cohorts), (c) NLR cut-off value (≤ 2 versus > 2), (d) sample size (≥ 200 versus < 200 participants), and (e) methodological quality (NOS score ≥ 8 versus < 8), with complete stratification results presented (**Table 2**).

**Table 2:** Subgroup analysis of NLR for overall survival.

Subgroup	Number of studies	HR (95% CI)	P value	Heterogeneity	
				I^2^	Ph
Survival analysis					
Univariate	5	1.97(1.15,3.36)	0.01	80%	< 0.01
Multivariate	5	2.44(1.39,4.28)	< 0.01	64%	0.03
Region					
Asian	6	2.26(1.24,3.46)	< 0.01	70%	< 0.01
Non-Asian	4	1.95(0.89,4.28)	0.09	76%	0.04
Cut-Off value					
≤ 2	6	2.08(1.09,3.99)	0.03	88%	< 0.01
> 2	4	2.23(1.47,3.36)	< 0.01	54%	0.09
Sample size					
< 200	7	2.36(1.21,4.61)	0.01	86%	< 0.01
≥ 200	3	1.85(1.11,3.07)	0.02	78%	0.01
NOS score					
< 8	6	2.04(1.29,3.25)	< 0.01	76%	< 0.01
≥ 8	4	2.41(1.18,4.90)	0.02	72%	0.01

It is noteworthy that although significant residual heterogeneity was observed across all study subgroups (all I² > 50%), the subgroup with a cut-off > 2 exhibited moderate heterogeneity (I² = 54%, P = 0.09), indicating relatively consistent and reliable conclusions regarding the association between NLR and OS across studies within this subgroup (HR = 2.23, 95% CI: 1.47-3.36, P < 0.01). This finding significantly strengthens the credibility of the association identified in this population.

However, no significant prognostic association was observed in the non-Asian subgroup (HR = 1.95, 95% CI: 0.89–4.28, P = 0.09), a result that may be attributed to the limited sample size (550 patients) and consequent insufficient statistical power. The direction and magnitude of the prognostic associations remained highly consistent across other subgroups: an elevated NLR consistently predicted poorer survival outcomes (P < 0.05). The overall subgroup differences are robust, and the non-significant result in the non-Asian population is likely a false negative due primarily to small sample size and low statistical power, rather than a true absence of association. Therefore, the effect in non-Asian populations requires further validation in larger studies.

### 3.4 Prognostic value of lymphocyte-to-monocyte ratio/monocyte-to-lymphocyte ratio

**Figure 7:**
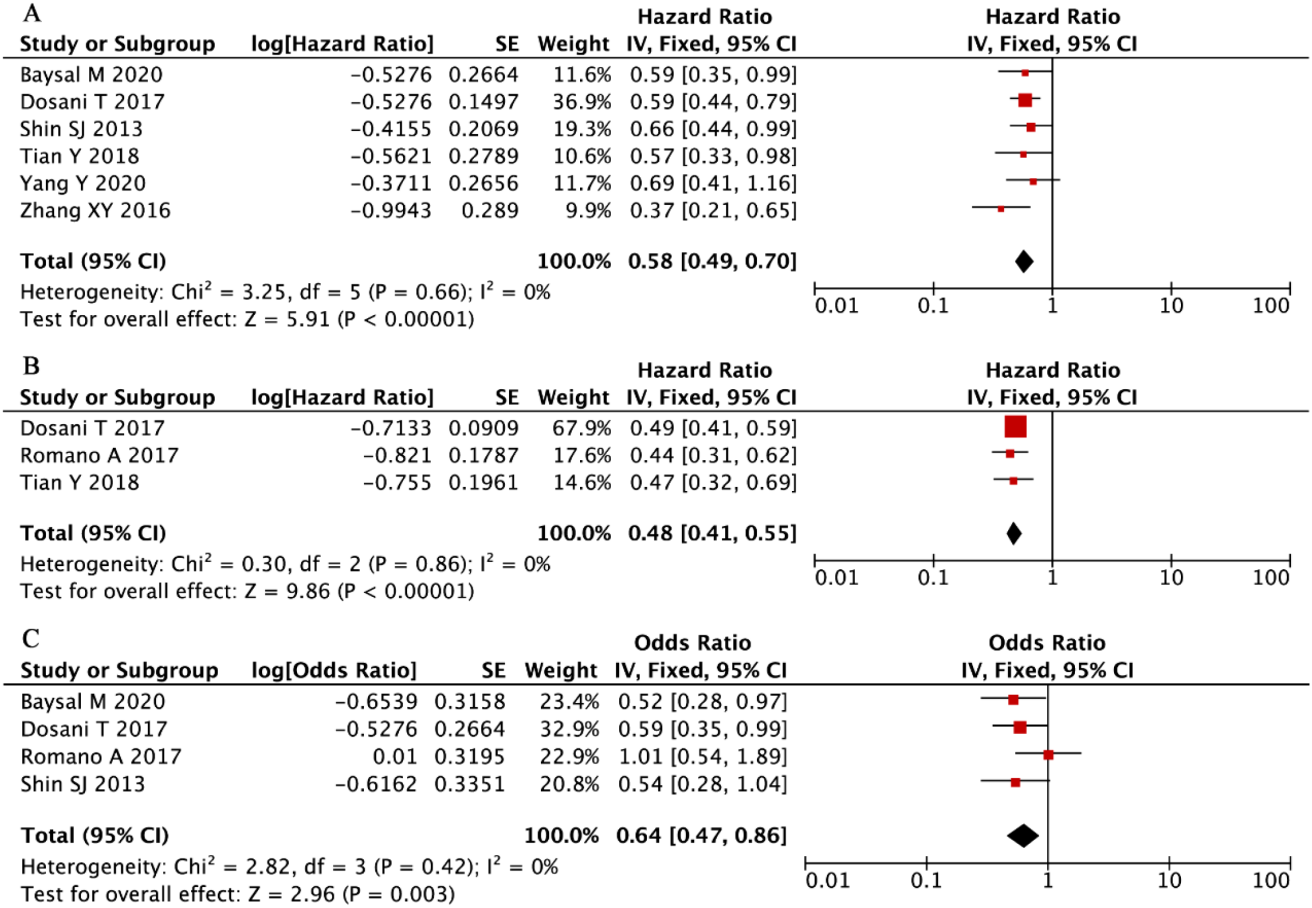
Forest plots summarizing the associations of LMR with **(A)** overall survival (OS), **(B)** progression-free survival (PFS), and **(C)** disease stage (ISS).

Our systematic evaluation of seven independent cohorts comprising 1,481 multiple myeloma patients (34, 42, 44, 45, 49, 55), established a statistically significant inverse relationship between elevated LMR and overall mortality risk. The fixed-effects meta-analysis demonstrated a pronounced survival benefit associated with higher LMR values (HR = 0.58, 95% CI: 0.49-0.70, P < 0.001, **Figure 7A**), with no evidence of between-study heterogeneity (I² = 0%, P = 0.66). These robust findings confirm that increased LMR serves as a consistent, reproducible predictor of superior OS in MM patients, with remarkable consistency observed across all investigated study populations and clinical settings.

Our integrated analysis of three clinical cohorts (n = 865 MM patients) (34, 39, 42), established a robust association between elevated LMR and superior PFS outcomes (HR = 0.48, 95% CI: 0.41-0.55, P < 0.001, **Figure 7B**), with complete homogeneity across studies (I² = 0%, P = 0.86). Complementing these findings, evaluation of four additional studies (n = 949) (34, 39, 49, 55), demonstrated that higher LMR values were significantly predictive of earlier disease stages at presentation (ISS III vs I-II: OR = 0.64, 95% CI: 0.47-0.86, P = 0.003, **Figure 7C**), with similarly negligible heterogeneity observed (I² = 0%, P = 0.42). These concordant results across distinct clinical endpoints - spanning both survival outcomes and disease severity metrics - provide compelling evidence for LMR’s utility as a consistent and reliable prognostic indicator in multiple myeloma management.

Our focused evaluation of a distinct cohort comprising 560 patients with multiple myeloma (61% male) (40), revealed that an elevated monocyte-to-lymphocyte ratio (MLR) was a significant predictor of adverse clinical outcomes, demonstrating strong associations with both reduced OS (HR = 1.45, 95% CI: 1.19-1.77, P < 0.001) and inferior PFS (HR = 1.52, 95% CI: 1.20-1.90, P < 0.001). These results not only validate but also extend prior epidemiological evidence, collectively establishing that both LMR and its reciprocal MLR represent clinically meaningful and complementary prognostic biomarkers in MM pathogenesis and disease progression. The robust consistency of these inverse relationships across multiple survival endpoints underscores the fundamental role of monocyte-lymphocyte homeostasis in MM pathophysiology and clinical outcomes.

### 3.5 Prognostic value of platelet-to-lymphocyte ratio

**Figure 8:**
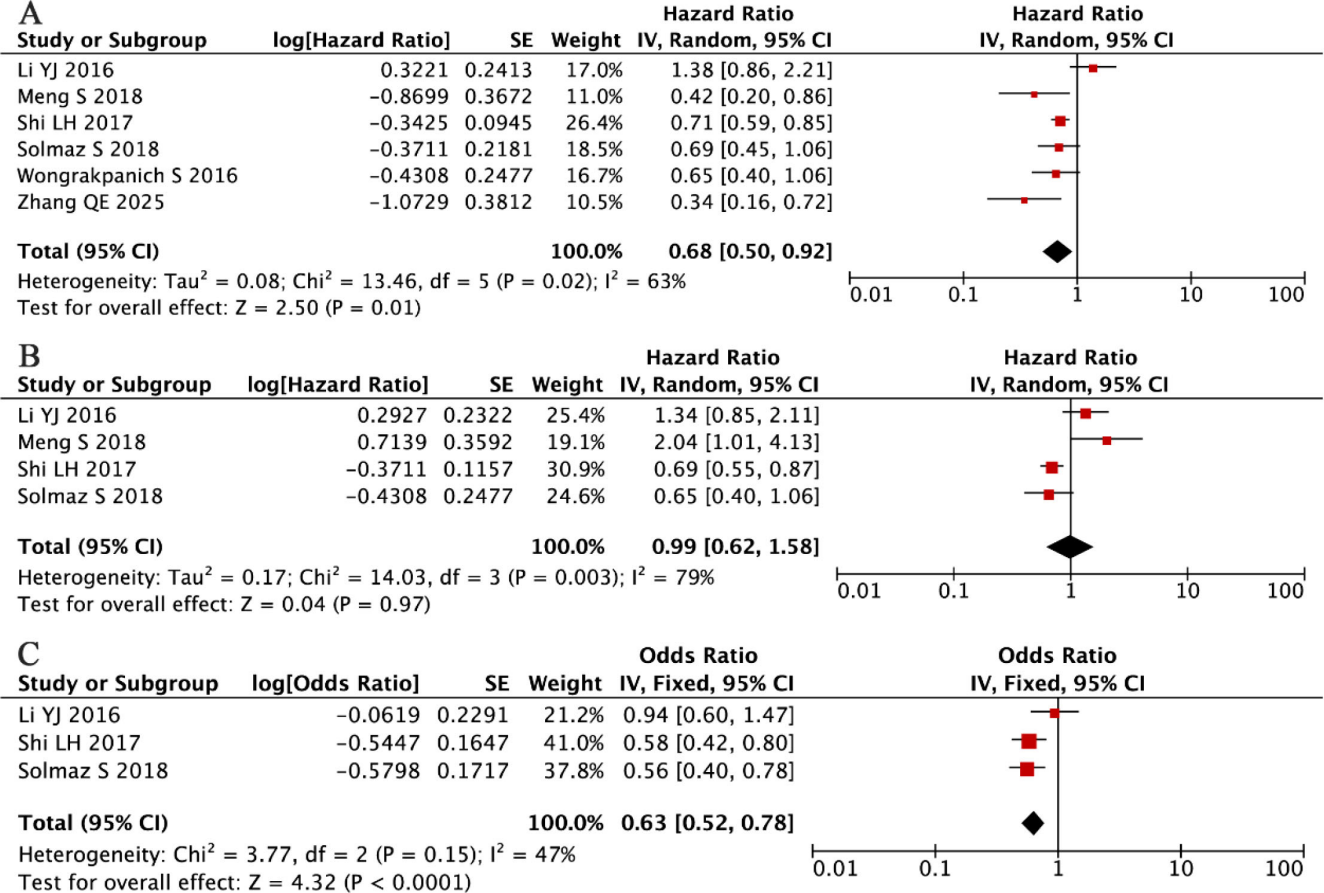
Forest plots summarizing the associations of PLR with **(A)** OS, **(B)** PFS, and **(C)** ISS.

Our comprehensive meta-analysis of six independent cohorts (total n = 1,510) (33, 36, 40, 41, 44, 56), evaluating the prognostic value of PLR in MM, revealed substantial between-study heterogeneity (I² = 63%, P = 0.02), necessitating the application of a random-effects model. The analysis results indicated that elevated PLR was associated with longer OS (HR = 0.75, 95% CI: 0.54-1.05, P = 0.01; **Figure 8A**).

Complementing these results, our pooled analysis of four additional studies (n = 1,227) (33, 36, 40, 41) found no significant association between PLR levels and PFS (random-effects model: HR = 0.99, 95% CI: 0.62-1.58, P = 0.67; **Figure 8B**), with significant residual heterogeneity (I² = 73%, P = 0.005).

Our investigation into the relationship between PLR and clinicopathological features in MM yielded noteworthy findings. A pooled analysis of three independent cohorts comprising 1,061 MM patients (36, 40, 41) revealed a statistically significant inverse association between elevated PLR levels and advanced disease stage (fixed-effects model: HR = 0.63, 95% CI: 0.51-0.78, P < 0.001; **Figure 8C**). This association was observed despite moderate between-study heterogeneity that did not reach statistical significance (I² = 47%, P = 0.15). The direction and magnitude of this effect remained consistent across all included studies, suggesting a potential role for PLR in disease stratification, although its prognostic value for survival outcomes appears limited based on our previous analyses.

### 3.6 Prognostic value of red cell distribution width

**Figure 9:**
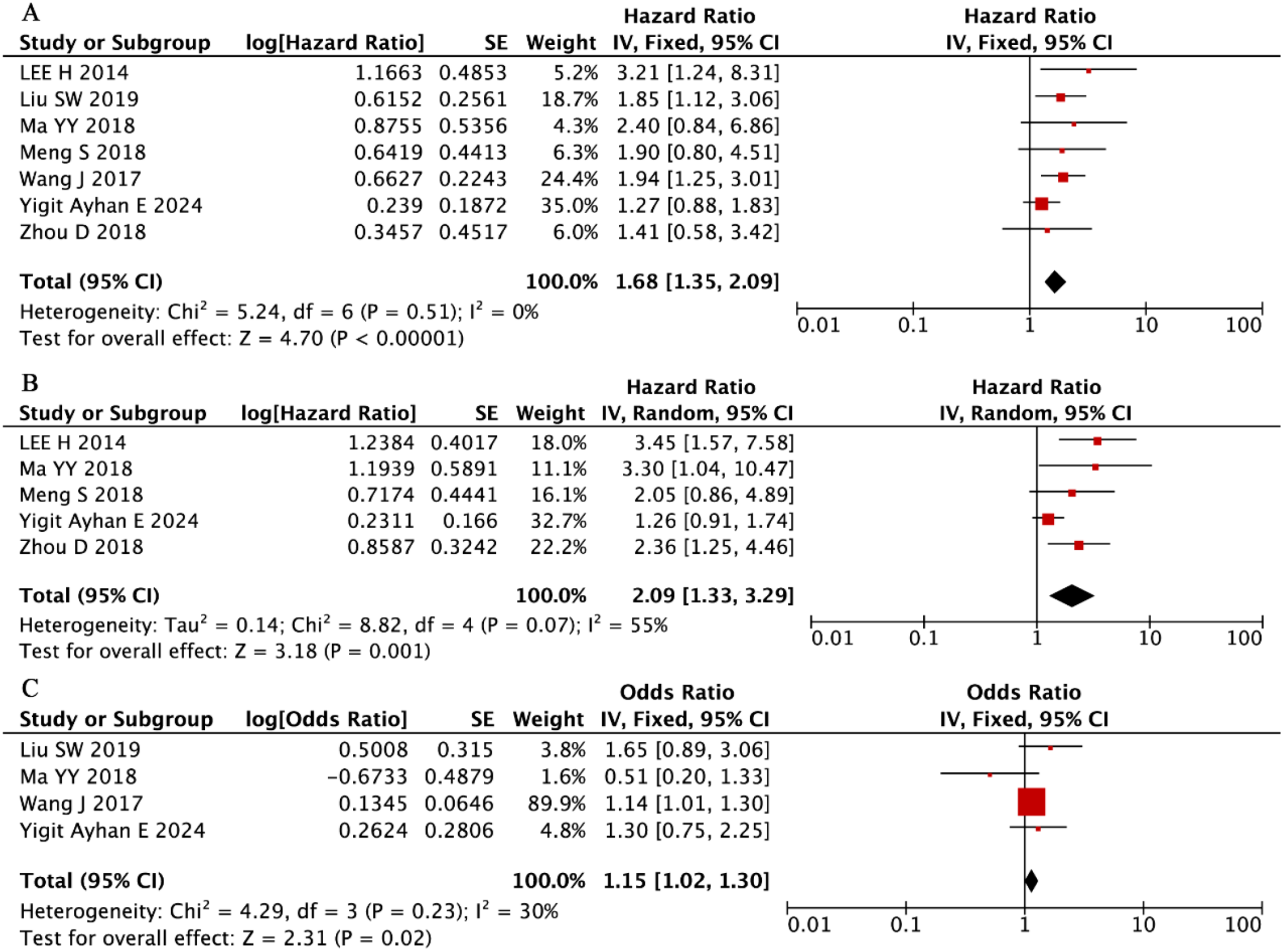
Forest plots summarizing the associations of RDW with **(A)** OS, **(B)** PFS, and **(C)** ISS.

Our systematic evaluation of seven independent cohorts comprising 1,141 MM patients (56% male) (33, 37, 43, 50, 51, 53, 54) established a robust association between elevated RDW levels and significantly worse OS outcomes (pooled HR = 1.68, 95% CI: 1.35-2.09, P < 0.001; **Figure 9A**). This clinically important relationship demonstrated remarkable consistency across all included studies, as evidenced by the complete absence of between-study heterogeneity (I² = 0%, P = 0.51). These findings position RDW as a reliable and reproducible prognostic hematologic marker in MM, with elevated values consistently predicting inferior survival irrespective of potential confounding factors or study-specific characteristics.

Our integrated analysis of five clinical cohorts encompassing 770 MM patients (56% male) (33, 43, 50, 51, 53) revealed that that elevated RDW values were associated with inferior PFS outcomes (random-effects model: HR = 2.09, 95% CI: 1.33-3.29, P = 0.001; **Figure 9B**). Although moderate heterogeneity was observed (I² = 55%, P = 0.07), the direction and magnitude of this association remained consistent all included studies.

Complementing these findings, the evaluation of four additional studies (n = 667) (37, 43, 53, 54) demonstrated that higher RDW levels were independently associated with more advanced disease stage at diagnosis (ISS III vs. I-II: OR = 1.15, 95% CI: 1.02-1.30, P = 0.02; **Figure 9C**) under a fixed-effects model framework. This clinically relevant relationship showed minimal heterogeneity (I² = 30%, P = 0.23), reinforcing RDW’s role as a robust biomarker for both disease progression and initial tumor burden assessment in MM patients.

### 3.7 Prognostic value of RDW-to-platelet ratio

Our systematic review identified a paucity of high-quality evidence regarding the prognostic utility of RPR in MM, with only two studies meeting the inclusion criteria (32, 57). The first investigation (n = 110; 53% male; median age = 61 years) (32) paradoxically reported a marked survival benefit associated with elevated RPR (HR = 0.037, 95% CI: 0.002-0.887, P = 0.03). In striking contrast, a subsequent study (n = 145; 54% male; median age = 59 years; optimal cutoff = 0.12) (57) identified elevated RPR as an independent predictor of poor prognosis, showing significant associations with both reduced PFS (HR = 3.30, 95% CI: 1.04-10.48, P = 0.043) and shorter OS (HR = 3.39, 95% CI: 1.90-5.94, P < 0.001).

These fundamentally opposed findings, together with the limited cumulative sample size (N = 255) and scarcity of eligible studies, preclude any definitive conclusions regarding RPR’s prognostic relevance in MM. The observed inconsistencies likely arise from methodological differences in RPR measurement, variability in patient characteristics, and heterogeneity in treatment protocols. Collectively, this evidence gap highlights the urgent need for large-scale, prospective, multicenter studies employing standardized RPR assessment and rigorous clinical correlation to clarify its potential role in MM risk stratification and outcome prediction.

### 3.8 Prognostic value of hemoglobin-to-RDW ratio

Our systematic evaluation identified only one eligible study assessing the prognostic relevance of HRR in MM, which included 180 patients (48% male; median age = 67 years) (55) using a predefined HRR cutoff of 0.61. This study established a statistically significant association between reduced HRR levels and unfavorable clinical outcomes (HR = 2.08, 95% CI: 1.31-3.03, P = 0.002).

Although current evidence is constrained by the single-study design and limited cohort size (N = 180), the well-established prognostic role of HRR across a broad spectrum of hematologic and solid tumors lends strong biological plausibility to its potential clinical relevance in MM. This preliminary but encouraging finding warrants confirmation through adequately powered, multicenter prospective studies employing standardized HRR quantification and detailed clinical correlation. Such validation would be essential to determine whether HRR can be integrated into future MM risk stratification models and guide individualized therapeutic decision-making.

## 4 Discussion

Multiple myeloma, a clinically heterogeneous plasma cell malignancy, requires prompt diagnostic evaluation and multimodal therapeutic intervention due to its intricate pathobiological mechanisms, which critically determine disease trajectory and prognosis. Contemporary research has elucidated the fundamental contribution of sustained inflammatory cascades in driving MM pathogenesis and progression, particularly through the tumor-promoting microenvironment (58).

The NLR, a well-established systemic inflammation marker derived from routine complete blood count parameters, has been validated as a robust prognostic indicator across diverse malignancies (59). Substantial evidence from clinical studies and meta-analyses consistently demonstrates the independent prognostic value of elevated pretreatment NLR in cancers such as cholangiocarcinoma (60), sepsis (61), colorectal cancer (62), squamous cell carcinoma of the penis (63), non-small cell lung cancer, gallbladder cancer (64), and diffuse large B-cell lymphoma (13). Our comprehensive meta-analysis confirms NLR’s significant prognostic utility in MM, with elevated levels strongly predicting inferior OS and PFS—findings that align with existing literature. Notably, the study by Li et al. (36) specifically established this association in elderly MM patients (≥ 65 years). Furthermore, our analysis revealed a significant correlation between increased NLR values and advanced International Staging System (ISS) classification. In the subgroup analysis of NLR and OS, patients were stratified using a cutoff value of 2, determined based on the median NLR threshold across the included studies. Patients with NLR > 2 exhibited significantly poorer prognoses compared with those having NLR ≤ 2. Our meta-analysis further supports that a higher NLR cutoff value is associated with worse survival outcomes, suggesting NLR > 2 may represent a potential threshold for identifying high-risk individuals. Collectively, these findings position NLR monitoring as a clinically valuable tool for dynamic disease assessment, offering critical supplementary information for therapeutic decision-making and risk stratification in MM management. The routine availability and cost-effectiveness of NLR measurement further enhance clinical practicality.

Tumor-associated macrophages, one of the most abundant immune cell populations in the tumor microenvironment, originate partly from local macrophage proliferation but predominantly from circulating monocytes recruited by tumor-derived chemokines (65). In addition, lymphocytes play a crucial role in regulating anti-tumor immune activity (66, 67). Our meta-analysis systematically evaluated the prognostic significance of the LMR, an established inflammatory marker, in MM patients. The findings demonstrated consistent associations between elevated LMR and improved OS and PFS, corroborating results from six previous studies (34, 39, 42, 48, 49, 55). Although Yang Y et al. (45) reported that the absolute lymphocyte count (ALC), but not LMR, served as an independent prognostic factor, this discrepancy did not substantially alter our pooled results. Notably, higher LMR values also showed significant correlation with earlier ISS stages, reinforcing its prognostic utility in MM. Complementary evidence from another study (40) confirmed that elevated MLR predicted inferior OS and PFS, collectively emphasizing the clinical relevance of leukocyte ratio–based prognostic markers in MM.

Elevated PLR reflects an imbalance between pro-inflammatory and anti-inflammatory responses within the tumor microenvironment, a phenomenon known to promote tumorigenesis and correlate with poor outcomes in various malignancies (68–71). However, the prognostic significance of PLR in MM remains poorly characterized, with existing studies yielding inconsistent findings. Our meta-analysis indicates that peripheral blood PLR is significantly associated with improved OS in MM but shows no significant prognostic value for PFS. The heterogeneity in treatment regimens across the included study populations may account for the discrepant associations observed with PLR. These findings underscore the need for larger prospective studies to clarify the potential prognostic and clinical utility of PLR in MM.

RDW reflects heterogeneity in erythrocyte volume (72). Elevated RDW levels have been associated with increased circulating cytokines, including interleukin-6 (IL-6) and tumor necrosis factor-α (TNF-α) (73), both of which are key inflammation-related mediators. RDW has been recognized as a powerful prognostic marker across multiple diseases and malignancies (74–76), including pancreatic (77), colorectal (78) and breast cancers (23). Our meta-analysis corroborates these observations in MM, demonstrating that elevated RDW significantly predicts inferior OS and PFS and is strongly associated with advanced ISS stages.

Platelet count is routinely measured in clinical practice, and increased platelet count is common in many cancers (79), where it has been linked to adverse outcomes. Several studies have shown that an elevated RPR is associated with poor prognosis in chronic hepatitis, pancreatitis, and acute myocardial infarction (80–82). Our meta-analysis identified only two studies examining RPR in MM, yielding contradictory results that preclude definitive conclusions. This substantial knowledge gap highlights the necessity for future large-scale investigations to clarify the potential prognostic relevance of RPR in MM.

Although the prognostic significance of hemoglobin (Hb) and RDW in cancer has been well-documented, both parameters are nonspecific and may be influenced by various non-neoplastic conditions including autoimmune disorders, hematologic diseases, and systemic inflammatory states. The HRR has recently emerged as a novel inflammatory biomarker with superior sensitivity and specificity compared to its individual components, reflecting systemic oxidative stress and inflammation more comprehensively. Growing evidence supports HRR as a reliable prognostic indicator across multiple malignancies (83), particularly in small cell lung cancer (84), hepatocellular carcinoma (85), nasopharyngeal carcinoma (86) and non-small cell lung cancer (87). However, its prognostic relevance in MM remains largely unexplored. Although the included study suggested an association between low HRR and adverse outcomes, the limited sample size (n = 180) precludes firm conclusions. This highlights the critical need for larger, well-designed clinical studies to validate HRR’s potential role in MM prognosis assessment, treatment response evaluation, and long-term survival prediction.

Several important limitations should be acknowledged when interpreting our meta-analysis findings. First, despite comprehensive sensitivity analyses and meta-regression adjustments for multiple confounding factors (including cutoff thresholds, cohort sizes, geographic distributions, and Newcastle–Ottawa Scale quality scores), significant residual heterogeneity persisted in the NLR and PLR analyses. Second, the predominance of retrospective designs among the included studies introduces potential selection and information biases, thereby limiting the overall strength of evidence. Third, our evaluation was constrained to basic clinicopathological parameters (sex and ISS classification) and did not incorporate critical prognostic determinants such as cytogenetic abnormalities and renal function indicators.

Methodologically, the variability in optimal cutoff values across studies and the extraction of hazard ratios from Kaplan–Meier curves in some cases may have reduced measurement precision. Furthermore, the limited number of studies assessing RPR and HRR prevented meaningful pooled analyses. Future large-scale, multicenter prospective studies employing standardized protocols and comprehensive data collection are warranted to validate these findings and further elucidate the prognostic significance of hematologic indices in MM.

In addition to well-established cytogenetic abnormalities such as del(17p), t (4,14), and t (14,16), which are recognized as markers of poor prognosis in multiple myeloma (MM), systemic inflammatory markers such as NLR, LMR, and RDW may offer complementary prognostic information by reflecting the host immune status and tumor–microenvironment interactions. Unlike cytogenetic alterations that primarily capture intrinsic tumor biology, hematologic ratios are dynamic and may better represent the systemic inflammatory burden and immune dysfunction contributing to disease progression and treatment resistance. Moreover, inflammation-based ratios such as NLR, LMR, and HRR simultaneously capture both pro-tumor and anti-tumor forces, providing deeper insights into the systemic immune-inflammatory responses driven by tumors. Moreover, ratio-based indicators are less susceptible to transient physiological fluctuations (e.g., infection, dehydration, or stress), making them more stable and reflective of chronic pathological immune activation compared with single-parameter biomarkers.

In summary, our meta-analysis provides compelling evidence that hematologic indices—particularly NLR, LMR, and RDW—serve as robust prognostic biomarkers for survival outcomes in MM, while the clinical significance of PLR and RPR warrants further investigation. These routinely available, cost-effective parameters offer substantial clinical utility for practical risk stratification and treatment optimization, potentially altering disease progression trajectories. Importantly, the prognostic value of these fundamental hematologic markers remains highly relevant in the context of modern MM management, demonstrating remarkable consistency despite evolving diagnostic and therapeutic landscapes.

## 5 Conclusion

NLR, LMR, and RDW represent promising prognostic biomarkers in multiple myeloma, with elevated NLR and RDW and decreased LMR consistently associated with poorer clinical outcomes. The prognostic significance of PLR, RPR, and HRR remains to be clarified and warrants further investigation. Given their accessibility and cost-effectiveness, these hematologic indices hold potential value for enhancing clinical risk stratification and guiding individualized therapeutic decision-making in MM.

## Data Availability

The original contributions presented in the study are included in the article/supplementary material. Further inquiries can be directed to the corresponding authors.
